# Procalcitonin Guiding Antimicrobial Therapy Duration in Febrile Cancer Patients with Documented Infection or Neutropenia

**DOI:** 10.1038/s41598-018-19616-3

**Published:** 2018-01-18

**Authors:** Hanine El Haddad, Anne-Marie Chaftari, Ray Hachem, Majd Michael, Ying Jiang, Ammar Yousif, Sammy Raad, Mary Jordan, Patrick Chaftari, Issam Raad

**Affiliations:** 1 Infectious Diseases, Infection Control & Employee Health, University of Texas MD Anderson Cancer Center, Houston, TX USA; 2 Emergency Medicine, University of Texas MD Anderson Cancer Center, Houston, TX USA

## Abstract

In this analysis, we identified febrile cancer patients with documented infections or neutropenia, whose procalcitonin levels are low at baseline or decrease on antibiotics. These patients had similar outcomes in terms of mortality and relapse of infection regardless of the duration of antimicrobial therapy (less or more than 7 days).

## Introduction

Clinical decisions are often complex and require serial patient evaluations to assess therapeutic responses and ensure favorable patient outcomes.

The management of febrile cancer patients could be particularly challenging. These patients frequently do not present with typical clinical syndromes and have multiple simultaneous sources of infectious and non-infectious causes of fever.

Because of their immunocompromised status due to the malignancy itself and treatment related toxicities, an empiric antimicrobial therapy is often begun in cancer patients presenting with fevers leading to a significant cost expenditure particularly in hospitalized patients^1^. However, increased antimicrobial use is associated with unwanted adverse effects and emergence of antimicrobial resistance leading to poor outcomes including increased mortality^2,3^. Serologic biomarkers might be useful adjunctive markers to guide antibiotic therapy. Procalcitonin (PCT) is a hormokine that has been studied in the general population to guide antimicrobial duration in the intensive care unit (ICU) as well as initiation of antibiotics in lower respiratory tract infections^4–6^. However, these studies excluded immunocompromised patients. Through several observational studies we have demonstrated that Procalcitonin could be a useful prognostic biomarker in febrile cancer patients with documented infections. However we did not study the role of PCT in guiding the duration of antimicrobial therapy^7,8^.

The objective of this study is to compare the outcomes of selected febrile cancer patients tested for procalcitonin based on their antimicrobial therapy duration. The patients were selected based on their PCT levels to help explore the applicability of a PCT based algorithm in guiding antimicrobial duration in cancer patients and febrile neutropenia.

## Methods

This is study is a post-hoc analysis of a prospective observational cohort conducted at a tertiary care center between July 2009 and July 2010.

Febrile cancer patients admitted to MDACC with available PCT levels were eligible for the study.

PCT was measured in the research laboratory using the Kryptor bioanalyser via an immunofluorescent assay with a lower detection limit of 0.075ng/mL. The PCT results were not available to the treating physician and did not therefore dictate the course of the antibiotics.

For every febrile patient, PCT levels were obtained on residual patient sera upon their initial presentation to the hospital for fever and subsequently, at days 4 to 7.

We included in this study febrile cancer patients who had clinically or microbiologically documented infection or presented with febrile neutropenia with no obvious source of infection. In addition, and based on cutoffs used in prior studies^4^, patients had to meet PCT response definition as follows: a PCT level less than 0.25 ng/ml on days 4–7 or a PCT level that has dropped by 80% on days 4–7 from baseline. Patients who received a short course of IV antibiotic of 7 days or less (group A) were compared to those who received a longer course with an antibiotic duration of more than 7 days (group B).

Categorical variables were compared using Chi-square or Fisher’s exact test, as appropriate. Continuous variables were compared using Wilcoxon rank-sum test. All tests were two-sided tests with a significance level of 0.05. The data analyses were performed using SAS version 9.3 (SAS Institute Inc., Cary, NC).

## Results

From July 2009 until June 2011, we identified 575 febrile cancer patients with available PCT levels that presented to the Emergency department or were admitted to MD Anderson Cancer Center.

Of the 196 patients who met the PCT response criteria, 121 had either febrile neutropenia or a documented infection and received antimicrobial treatment. These patients were further divided in two groups based on their antibiotic therapy duration: 7 days or less (group A) versus more than 7 days (group B).

The baseline characteristics of the study population were comparable among the 2 groups in terms of sex, age, type and stage of malignancy, as well as rate of neutropenia (absolute neutrophil count (ANC) < 500) and active cancer therapy (Table 1).

**Table 1:** Comparison between patients with PCT response with different IV antibiotic durations.

Variables	Short IV antibiotic treatment ( ≤ 7 days) (n = 44) N (%)	Long IV antibiotic treatment ( > 7 days) (n = 77) N (%)	*p*-value
**Age (years), median (range)**	55 (5–79)	58 (9–79)	0.39
**Gender, male**	23 (52)	41 (53)	0.92
**Underlying cancer**			0.50
Hematologic malignancy	36 (82)	59 (77)	
Solid tumor	8 (18)	18 (23)	
HSCT	10 (23)	23 (30)	0.40
Type of HSCT			>0.99
Autologous	2/10 (20)	5/23 (22)	
Allogeneic	8/10 (80)	18/23 (78)	
**Status of cancer**			0.77
Active	38 (86)	65 (84)	
Remission	6 (14)	12 (16)	
**For patients with active cancer**			0.81
New diagnosis	16/38 (42)	25/65 (38)	
Relapsed	12/38 (32)	19/65 (29)	
Refractory/progression/Metastasis	10/38 (26)	21/65 (32)	
**Neutropenia at onset**	28 (64)	52 (68)	0.66
**Active treatment for cancer**	40 (91)	67 (87)	0.52
**Micobiologic positivity**	23 (52)	37 (48)	0.66
Source of infection			
Bacteremia	8 (18)	18 (23)	0.50
Respiratory	8 (18)	14 (18)	> 0.99
GU	10 (23)	7 (9)	0.06
Intraabdominal	3 (7)	6 (8)	> 0.99
SSTI	2 (5)	6 (8)	0.71
CNS	0 (0)	1 (1)	> 0.99
No source	13 (30)	25 (32)	0.74
**Fungal Infection**	4 (9)	4 (5)	0.46
**ICU admission**	6 (14)	13 (17)	0.64
**Duration of IV antibiotic treatment (days), median (range)**	5 (3–7)	15 (8–50)	
**Defervescence**	44 (100)	77 (100)	
**Duration between fever onset and defervescence (days), median (range)**	2 (1–6)	2 (0–16)	0.59
**Defervescence within 72 hours of fever onset**	36 (82)	55 (71)	0.20
For patients with microbiological positivity			
Microbiology eradication	13/14 (93)	25/28 (89)	> 0.99
Duration between date of positive culture and microbiological eradication (days), median (range)	1 (0–5)	3 (0–9)	0.07
Microbiology eradication within 72 hours of	9/14 (64)	13/28 (46)	0.27
**Relpase within one month**	0	0	
**Deep-seated infection**	5 (11)	9 (12)	0.96
**Death within one month**	0 (0)	1 (1)	> 0.99
**Infection-related death within one month**	0	0	

The sites of infection were also comparable among both groups. Half the patients had microbiologically documented infections. The most common sources of infection were bacteremia (18 vs 23%) and pneumonia (18% in both groups). No identifiable source was documented in 30% and 32% of groups A and B respectively (Table 1).

The outcomes were similar among both groups including resolution of the infection, rate of relapse, infection related mortality and 30 day- mortality, regardless of the antimicrobial duration received. They were also similar in terms of microbiologic eradication and time to defervescence (Table 1).

## Discussion

Our study shows that a selected group of febrile cancer patients, particularly those with a low baseline PCT or a PCT that decreases with appropriate therapy might not require prolonged antimicrobial therapy beyond 7 days. These patients have similar rates of infection resolution, relapse and mortality compared with those receiving a prolonged course of antimicrobial therapy. This is the first study exploring the use of procalcitonin as a theragnostic marker in febrile cancer patients with documented infections or neutropenia.

A specific challenge to the cancer population is the elevation in inflammatory markers driven by the tumor and mucositis^8,9^. However, previous studies in the cancer population, showed that PCT levels were higher in cancer patients with fever compared to those without fever. In addition, PCT levels were the highest in febrile cancer patients with sepsis or bacteremia^8^. Furtheremore, in patients with suspected or documented bacterial infections, PCT levels decreased on appropriate therapy^10^.

Another important aspect is the potential role of PCT in guiding antimicrobial therapy duration in febrile neutropenia. The practice of keeping cancer patients, particularly neutropenic patients on prolonged duration of antimicrobials is based on literature going back to 1969. During that time, Pizzo *et al*.^11^ showed a higher rate of recurrent fevers when antimicrobial therapy was discontinued before the resolution of neutropenia in patients with no clear source of infection. In this particular group, laboratory markers may have a role in identifying patients who could safely receive a brief duration of therapy while simultaneously limiting unnecessary extended course of antimicrobial therapy. One small randomized controlled trial of PCT use for antimicrobial PCT duration in low risk febrile neutropenia failed to achieve significant reduction in antimicrobial therapy duration^12^. However, this may not apply to high risk profoundly neutropenic patients who are exposed to extensive antimocrobials for a prolonged duration.

Our study has several limitations: the sample size is small and the analysis is post-hoc, making specific subgroups related analyses statistically difficult. The study does not take into account the severity of illness of patients in both groups. In addition, PCT results were not made available to the treating physciain and it is unclear if more frequent PCT monitoring can further shorten the duration of antimicrobial therapy.

## Conclusion

PCT might be an adjunctive biomarker in identifying patients who could be candidates for a short duration of antimicrobial therapy particularly cancer patients with a non-infectious cause for the fever. PCT guided algorithm may limit the duration of antibiotics, reduce adverse events and prevent the emergence of antimicrobial resistance.

Large randomized controlled trials comparing procalcitonin guided antimicrobial therapy vs. standard of care to limit unnecessary exposure to antimicrobials in immunocompromised cancer patients are warranted.
