# Author Correction: Procalcitonin Guiding Antimicrobial Therapy Duration in Febrile Cancer Patients with Documented Infection or Neutropenia

**DOI:** 10.1038/s41598-018-24172-x

**Published:** 2018-04-19

**Authors:** Hanine El Haddad, Anne-Marie Chaftari, Ray Hachem, Majd Michael, Ying Jiang, Ammar Yousif, Sammy Raad, Mary Jordan, Patrick Chaftari, Issam Raad

**Affiliations:** 1 Infectious Diseases, Infection Control & Employee Health, University of Texas MD Anderson Cancer Center, Houston, TX USA; 2 Emergency Medicine, University of Texas MD Anderson Cancer Center, Houston, TX USA

Correction to: *Scientific Reports* 10.1038/s41598-018-19616-3, published online 18 January 2018

The original version of this Article contained a typographical error in the spelling of the author Majd Michael which was incorrectly given as Majd Micheal. This has now been corrected in the PDF and HTML versions of the Article.

